# Major increase in admission- and incidence rates of acute colonic diverticulitis

**DOI:** 10.1007/s00384-014-1888-9

**Published:** 2014-05-08

**Authors:** Aras Jamal Talabani, Stian Lydersen, Birger H. Endreseth, Tom-Harald Edna

**Affiliations:** 1 Department of Surgery, Levanger Hospital, North-Trondelag Hospital Trust, Nord-Trøndelag, Norway; 2 Regional Centre for Child and Youth Mental Health and Child Welfare – Central Norway, Faculty of Medicine, Norwegian University of Science and Technology, Trondheim, Norway; 3 Department of Gastroenterological Surgery, St Olavs Hospital, University of Trondheim, Trondheim, Norway; 4 Institute of Cancer Research and Molecular Medicine, Faculty of Medicine, Norwegian University of Science and Technology, Trondheim, Norway; 5 Unit for Applied Clinical Research, Institute of Cancer Research and Molecular Medicine, Faculty of Medicine, Norwegian University of Science and Technology, Trondheim, Norway

**Keywords:** Acute colonic diverticulitis, Epidemiology, Admission rates, Incidence rates, Incidence rate ratio

## Abstract

**Purpose:**

Hospitalization for acute colonic diverticulitis has become more and more frequent. We studied the changes in the rate of admission and incidence of the disease during the last 25 years.

**Methods:**

We performed a retrospective analysis of all cases treated for acute diverticulitis during 1988–2012 at one hospital serving a defined population in Mid-Norway. The study made a distinction between admission rates and incidence rates. The admission rates defined the total number of cases admitted, while the incidence rates defined the number of new patients hospitalized for acute diverticulitis (first admission). Poisson regression was used to analyse factors associated with diverticulitis incidence rates.

**Results:**

A total of 851 admissions in 650 different patients were identified, with an overall admission rate of 38.5 (CI 35.9 to 41.1) per 100,000 person-years. The admission rate increased from 17.9 (CI 14.1 to 22.3)/100,000 during 1988–1992 to 51.1 (CI 44.8 to 58.0)/100,000 during 2008–2012. Poisson regression analysis showed a significant increase in admission rates with a factor of 2.8 (C.I. 2.2 to 3.5) during 25 years. The overall incidence rate (IRR) of new patients was 29.4 (CI 27.1 to 31.7)/100,000 person-years. IRR increased significantly with a factor of 2.6 (CI 1.96 to 3.34) during 25 years, while IRR for perforations increased even more, by a factor of 3.3 (CI 1.24 to 8.58).

**Conclusion:**

The hospital admission rates as well as incidence rates for acute colonic diverticulitis increased significantly during the 25-year time span.

## Introduction

Diverticular disease of the colon occurs with increasing incidence in Western countries and places a burden on healthcare in these countries [1–6]. The prevalence is age dependent and increases from 5 % in the population aged 30–39 years to 60 % among those older than 80 years of age [7]. The disease most commonly involves the sigmoid and left colon, but can occur anywhere in the large intestine [4, 7, 8].

The majority of patients with colonic diverticulosis will be asymptomatic, but 4–25 % will experience acute or chronic symptomatic disease [8–13]. Acute inflammation is the most common complication of colonic diverticulosis [14]. In some patients, the inflammation leads to acute intestinal obstruction, but more often perforation of the colon with abscess formation and/or peritonitis, as described by Hinchey et al. in 1978 [15]. Late complications include colonic strictures and fistulas.

During the last decades, we have observed a subjective increase in the number of patients admitted to our department due to acute colonic diverticulitis. Geographical variation in the incidence rates of colonic diverticulosis, acute diverticulitis and the related complications is substantial [16–19], and we were unable to find relevant European epidemiologic studies on incidence of acute colonic diverticulitis during the same period. We conducted this study to assess the rate of admissions for acute colonic diverticulitis in our department and to evaluate potential changes in the incidence of this disease in our region.

## Patients and methods

All cases admitted to Levanger Hospital for acute colon diverticulitis during 25 years, between January 1988 and December 2012, were included in this series. The hospital is a first-line hospital serving the population of ten municipalities in North Trondelag county located in Mid-Norway. The patients were identified through the discharge diagnosis in the patient administrative system, using the Norwegian ICD-9 diagnosis codes 562.1 to 562.4 (1988–1998) and ICD-10 diagnosis codes K57.3 to K57.9 (1999–2012). In the presentation, the 25 years were categorized into five 5-year periods.

**Fig. 1:**
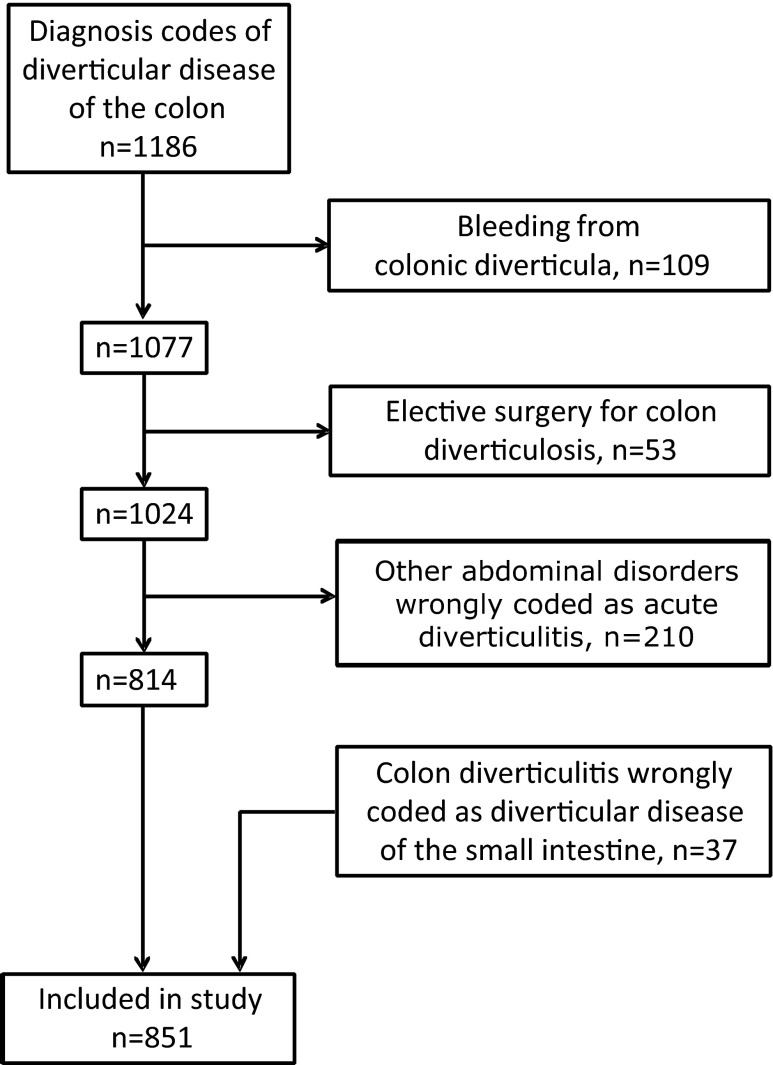
Flow chart for study. Cases diagnosed with acute colonic diverticulitis at Levanger Hospital 1988–2012

In all, 1,186 admitted cases were identified. Between November 2011 and February 2013, one gastrointestinal surgeon, who was employed at the department during the entire study period, retrospectively studied all medical records. The diagnoses were validated and a total of 851 admitted cases were included in the study. For an outline of included cases, see Fig. 1.

The diagnosis of colonic diverticulitis was based on clinical findings, temperature, C-reactive protein (CRP) and response to treatment. In 771 (91 %) of the cases, diagnosis was confirmed by radiology, endoscopy or an emergency operation. CRP was measured in g/L during the whole study period.

We classified the cases as uncomplicated or complicated acute colonic diverticulitis. Acute complicated diverticulitis was further subclassified into four categories: abscess formation (Hinchey stage I or II), bowel obstruction related to stenosis, perforation with purulent peritonitis (Hinchey stage III) or faecal peritonitis (Hinchey stage IV).

During the 25 years of study, some patients were admitted for acute colonic diverticulitis more than once. Each hospital stay for this disease counted as a new admission. An interval between admissions of at least 3 months counted as an admission due to recurrent diverticulitis, as suggested by Gervaz and Ambrosetti [20].

The study made a distinction between admission- and incidence rates. The admission rates define the total number of cases admitted for acute diverticulitis, while the incidence rates define the number of new patients hospitalized for acute diverticulitis (first admission). Both rates are given as the number of events in the specified population over a given time period, divided by the total person-time at risk during the period [21].

The medians of two samples were compared using the Wilcoxon’s test. The medians of more than two samples were compared using Kruskal-Wallis test. Poisson regression was used to analyse factors associated with incidence rates of acute diverticulitis. The incidence rate ratio (IRR) was defined as the ratio of two incidence rates. The analyses were adjusted for age (in 5-year intervals 20–24, 25–29 up to 95–99), gender and calendar year from 1988 to 2012. Nonlinear relationships were explored by using fractional polynomials [22]. The age and sex distribution of the ten municipalities around Levanger Hospital for every year between 1980 and 2012 was obtained from Statistics Norway. During the study period, the referral population of the hospital increased with 9.8 %, from 85,741 (43,072 males) in 1988 to 94,174 (47,117 males) in 2012, and the median age in males and females increased from 32.4 and 34.2 years to 38.9 and 38.9 years, respectively.

Time until recurrence (readmission at least 3 months after the primary admission) was assessed using Kaplan-Meier analysis.

Two-sided *p* values <0.05 were considered significant. Ninety-five percent confidence intervals (CI) are reported where relevant. Medians are reported with range (minimum to maximum) where relevant. The analyses were performed using SPSS 18 (SPSS Inc., Chicago, IL, USA) and STATA 12 (Stata Corp LP, College Station, TX, USA).

### Approvals

The Regional Committee for Research Ethics, Health Region 4 in Norway, approved the study.

## Results

### Admission rates

Over the 25-year study period, a total of 650 patients had 851 admissions for acute diverticulitis. There were 519 patients admitted once, 91 twice, 25 three times, nine four times, one five times, four six times and one ten times for acute diverticulitis.

**Fig. 2:**
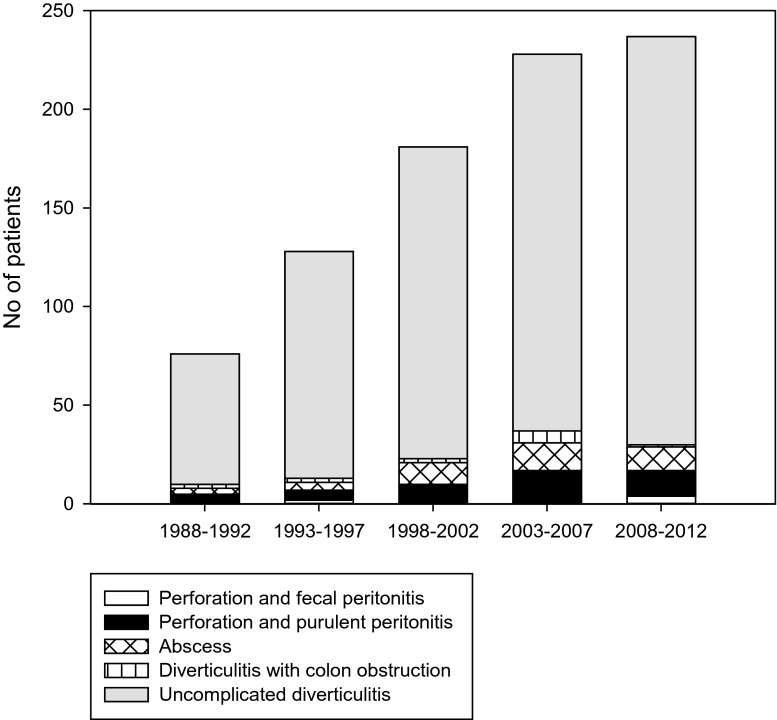
Cases admitted with acute diverticulitis per 5-year period in relation to different manifestations of diverticulitis

During the entire study period, we observed a continuous increase in cases of acute diverticulitis admitted to hospital, from 76 during the first 5-year period (1988–1992) to 237 cases during the last period (2008–12). Figure 2 illustrates the number of cases admitted per 5-year period in relation to different manifestations of acute diverticulitis.

The overall admission rate in the defined population was 38.5 (CI 35.9 to 41.1) per 100,000 person-years. It was 17.7 (CI 14.1 to 22.3)/100,000 during 1988–1992 and increased by each succeeding 5-year period to 29.4 (CI 24.5 to 35.0)/100,000, 41.6 (CI 35.8 to 48.1)/100,000, 51.0 (CI 44.6 to 58.1)/100,000 and 51.1 (CI 44.8 to 58.0)/100,000 during 2008–2012. The overall admission rate of patients with perforated acute diverticulitis with purulent or faecal peritonitis was 2.52 (CI 1.95 to 3.33)/100,000 person-years. It increased from 1.16 (CI 0.38 to 2.71)/100,000 during 1988–1992 to 3.66 (CI 2.13 to 5.87)/100,000 during 2008–2012. Using Poisson regression analysis to adjust for the effect of gender and age, the yearly increase in admission rates was 4.3 % (3.4 to 5.4) for acute diverticulitis and 5.7 % (1.8 to 9.8) for perforated diverticulitis, corresponding to 2.79, respectively, 3.80 times increase over 25 years.

**Fig. 3:**
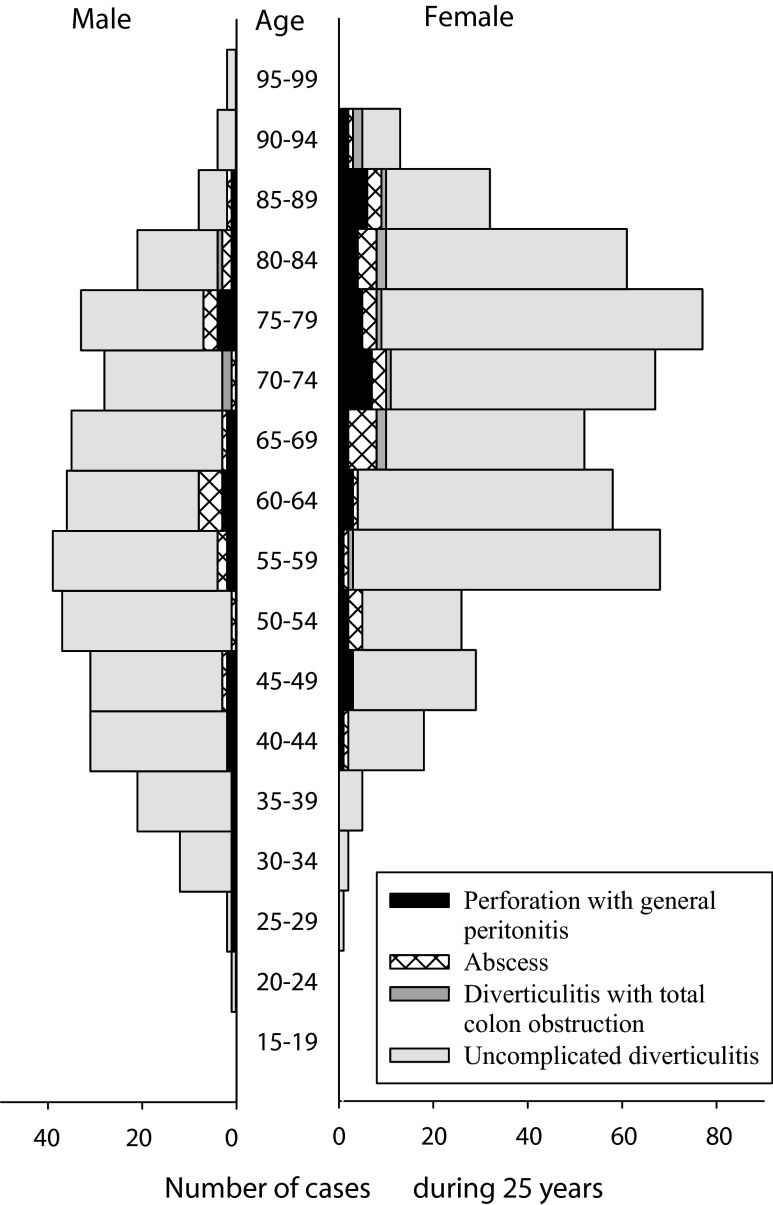
Number of cases admitted with acute diverticulitis during 25 years in relation to age and gender

There were 738 (86.7 %) admissions for uncomplicated and 113 (13.3 %) admissions for complicated acute diverticulitis. Forty-seven cases (5.5 %) had perforated diverticulitis with purulent peritonitis, 44 (5.2 %) had diverticulitis with an abscess, 13 (1.5 %) had acute diverticulitis with total colonic obstruction and nine (1.1 %) had perforated diverticulitis with faecal peritonitis. Figure 3 illustrates the distribution of age, gender and subtypes of acute diverticulitis among all cases included.

Eighty-nine percent (50/56) of the cases with perforated acute diverticulitis had their perforation during the first hospital stay. Three cases had the perforation at the second admission, while the last three cases perforated during the third, fourth and tenth admissions.

The basis for the diagnosis of acute diverticulitis changed throughout the study period. Clinical assessment included temperature and CRP, which were unchanged, but there was a notable change in the use of CT scans in the diagnostic process. Table 1 shows the maximum values of CRP during the stay for each 5-year period. The rate of patients undergoing an acute CT scan increased with time and was 6.6 % (5/76) during 1988–1992, 7.0 % (9/128) in 1993–1997, 15.4 % (28/182) in 1998–2002, 33.8 % (77/228) in 2003–2007 and 74.7 % (177/237) during 2008–2012.

**Table 1:** Highest value of C-reactive protein (CRP) measured during the stay in the cases treated for acute diverticulitis 1988–2012, median (min–max) during each 5-year period

	1988–92	1993–87	1998–2002	2003–07	2008–12	*p* value^a^
Uncomplicated diverticulitis (*n* = 738)						
CRP at admission	62 (1–191)	81 (1–452)	65 (0–353)	86 (4–287)	96 (2–316)	<0.001
Max CRP (mg/L)	83 (1–286)	96 (10–453)	107 (6–385)	112 (6–333)	126 (6–427)	0.003
Complicated diverticulitis (*n* = 113)						
CRP at admission	104 (33–197)	96 (26–329)	120 (0–620)	199 (6–391)	184 (5–393)	0.27
Max CRP (mg/L)	104 (33–197)	178 (26–329)	220 (5–620)	252 (6–509)	296 (5–528)	0.003

Values for CRP were available in 790 cases

^a^Kruskal-Wallis test

**Fig. 4:**
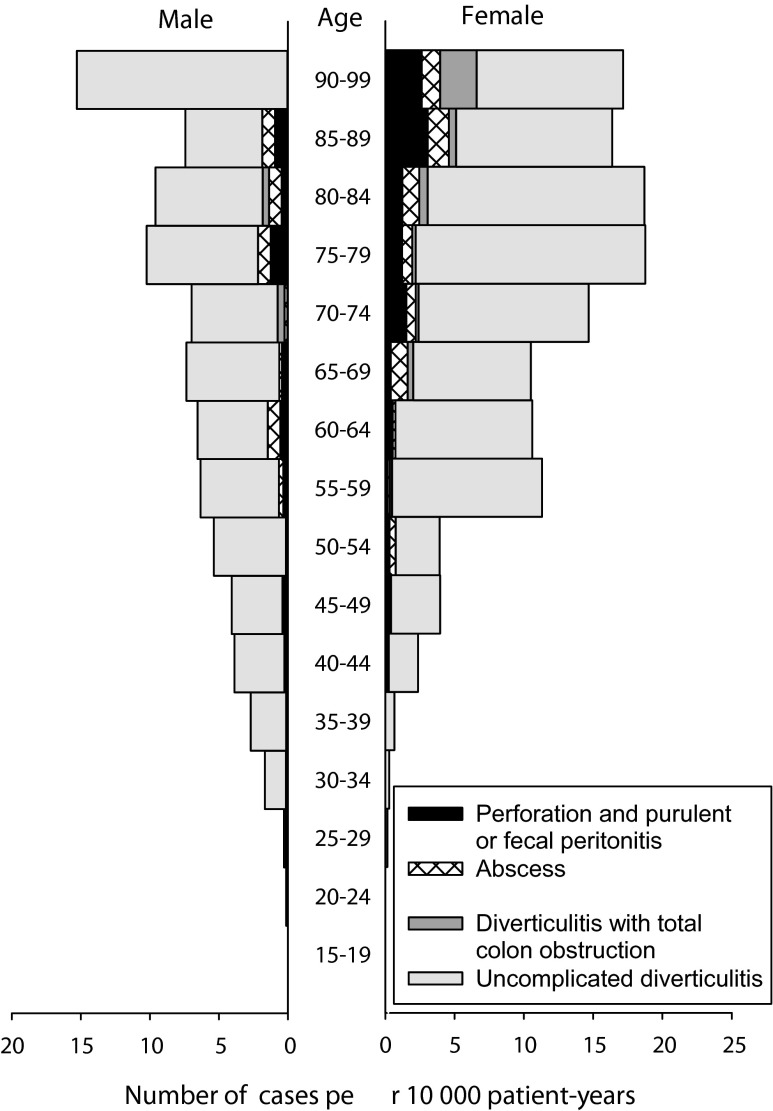
Number of cases admitted with acute diverticulitis per 10,000 person-years in relation to age and gender

Figure 4 shows the admission rate of acute diverticulitis per 10,000 person-years in relation to age and gender. Admissions for acute diverticulitis were male predominant up to 55 years, thereafter female predominant.

### Incidence rates

During the study period, 650 different patients (265 male and 385 female) were hospitalized due to acute diverticulitis. During each of the 5-year periods from 1988–92 to 2008–12, the number of patients were 67, 96, 136, 172 and 179, respectively. The overall incidence rate was 29.4 (CI 27.1 to 31.7)/100,000 person-years and increased over time. It was 15.6 (CI 12.1 to 19.8)/100,000 during 1988–1992 and increased by each succeeding 5-year period to 22.1 (CI 17.9 to 27.0)/100,000, 31.1 (CI 26.1 to 36.8)/100,000, 38.5 (CI 32.9 to 44.7)/100,000 and to 38.6 (CI 33.1 to 44.7)/100,000 during 2008–2012.

A Poisson regression analysis showed that for each calendar year, the IRR increased with 4.0 % in both genders, see Table 2. This corresponds to a 2.56-fold increase in incidence during 25 years (CI 1.96 to 3.34). For each 5-year increase in age, the IRR increased with 3.9 % in males and 5.1 % in females. Separate results for younger age and for perforations with peritonitis are also shown in Table 2. IRR for all 50 primary perforations was 1.050 (CI 1.009 to 1.094) per calendar year, corresponding to an increased perforation incidence of 3.26 times (CI 1.24 to 8.58) during 25 years.

**Table 2:** Factors associated with first acute diverticulitis incidence rate ratios (adjusted IRRs from Poisson regression 1988–2012)

	Male		Female	
	IRR (CI)	*p* value	IRR (CI)	*p* value
Diverticulitis, all (*n* = 650)				
Calendar year	1.040 (1.023 to 1.058)	<0.001	1.040 (1.025 to 1.055)	<0.001
Age (5-year intervals)	1.039 (1.032 to 1.046)	<0.001	1.051 (1.045 to 1.057)	<0.001
First perforation with peritonitis (*n* = 50)				
Calendar year	1.013 (0.952 to 1.079)	0.67	1.077 (1.0203 to 1.137)	0.007
Age (5-year intervals)	1.026 (1.001 to 1.056)	0.045	1.065 (1.042 to 1.089)	<0.001
Diverticulitis, <50 years of age (*n* = 112)				
Calendar year	1.061 (1.025 to 1.098)	0.001	1.040 (1.025 to 1.055)	<0.001
Age (5-year intervals)	1.083 (1.049 to 1.118)	<0.001	1.051 (1.045 to 1.057)	<0.001

**Fig. 5:**
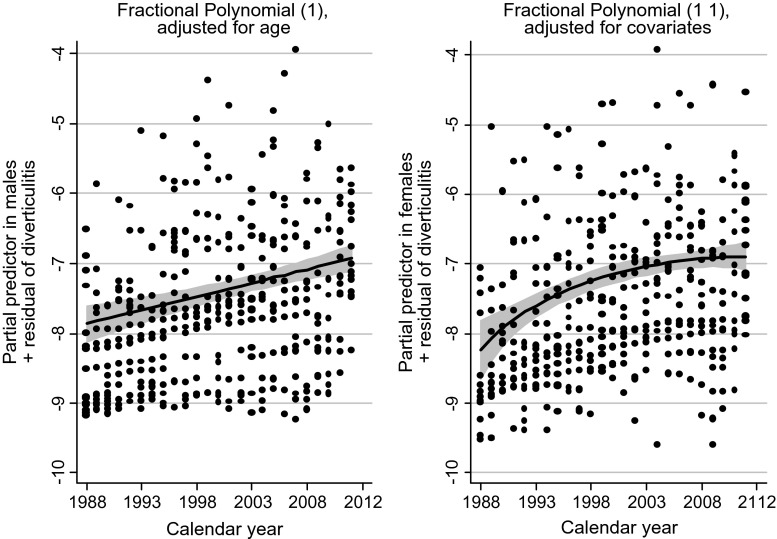
Effects of calendar year on the number of patients with their first admission for acute diverticulitis (logarithmic scale on the *y*-axis) in Poisson regression with fractional polynomials, males in the figure to the *left*. The effect of calendar year is linear for the male group, nonlinear for the female group, with an increase until it stabilizes around 2000–2005. Confidence intervals are *shaded grey*

**Fig. 6:**
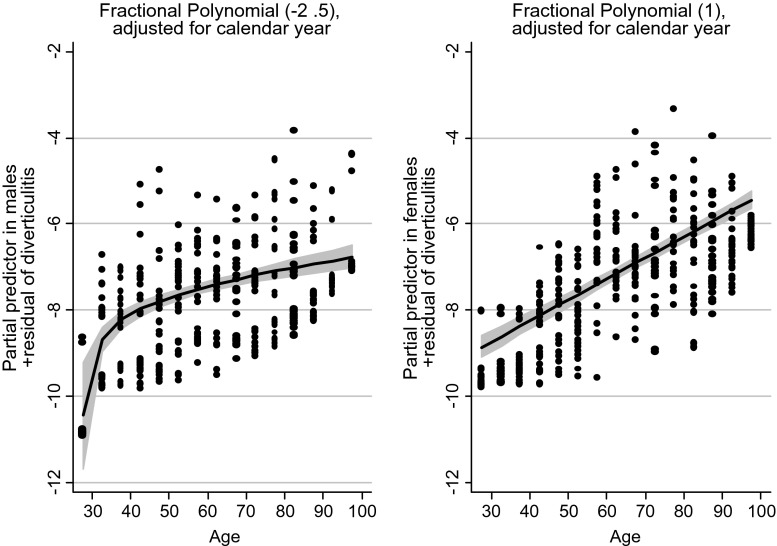
Effects of age on the number of patients with their first admission for acute diverticulitis (logarithmic scale on the *y*-axis) in Poisson regression with fractional polynomials, males in the figure to the *left*. The effect of age is linear for the female group, and nonlinear for the male group. Confidence intervals are *shaded grey*

Results of nonlinear analyses based on fractional polynomials after Poisson regression analysis of the two continuous variables calendar year and age separately are shown in Figs. 5 and 6. Up to 1992, the incidence was higher in males, but from 1993 to 2012, females dominated. Concerning age, the incidence was higher in males up to 55 years, thereafter females prevailed.

**Fig. 7:**
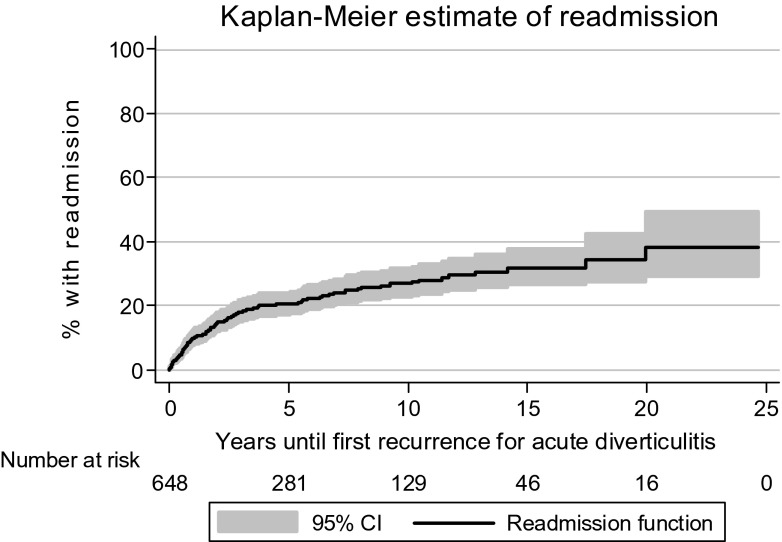
Kaplan-Meier estimate of years until the first recurrence for acute diverticulitis

Figure 7 shows the results of Kaplan-Meier analysis of time until the first recurrence for diverticulitis. In this analysis, 20 patients who had been readmitted during the first 3 months after the first episode were excluded. The analysis showed that after 1 year, the estimated recurrence rate was 7.0 % (CI 5.1 to 9.5), after 5 years, 18.0 % (CI 14.8 to 21.8) and after 10 years, 24.6 % (CI 20.5 to 29.3).

## Discussion

The purpose of this study was to evaluate potential changes in admission- and incidence rates of patients with acute diverticulitis during the 25 years of study. We found significant changes with a 2.9-fold increase in the admission rate and a 2.6-fold increase in the incidence rate.

The admission rates increased with age and by time (Table 2). However, the increase in females slowed during the later years, as can be seen in Fig 5. There was a male predominance among patients younger than 55 years, shifting to female predominance in the older age groups. The life expectancy of the Norwegian population is increasing, and the number of elderly patients in need for treatment of age-dependent diseases will continue to increase. This confirms that patients with acute colonic diverticulitis place increased burden on healthcare.

Throughout the study period, CT scans became more commonly used for patients hospitalized with acute abdominal conditions. In this series on patients with acute diverticulitis, CT was used for diagnosis in 7 % at the beginning and in 75 % towards the end of the 25 years. Thus, it seems likely that some patients with acute colonic diverticulitis could have been given an unspecified diagnosis of acute abdomen during the earlier years of the study.

However, during the first 10 years of this study, fewer patients were discharged from our hospital with an unspecified diagnosis of acute abdomen compared to the later 15 years. A Swedish study of 3,349 patients with acute abdomen showed that acute diverticulitis differed from nonspecific abdominal pain in both clinical presentation and laboratory investigations [23].

Very few have investigated the admission rates of all types of acute colonic diverticulitis. Two large investigations from USA [18, 24] studied hospital admissions for acute diverticulitis between 1998 and 2005. The rate of admission increased from 61.8/100,000 to 75.5/100,000 during the study period. These admission rates were even higher than found in the present study, which was 51.1/100,000 during 2008–2012. In patients managed in the USA from 2002 to 2007, the admission rate increased by 9.5 % [25]. Obesity, a more sedentary life and different diets may predispose one for acute diverticulitis [26], and these factors may in part explain the differences in incidence rates between different populations. The overall incidence of perforated diverticulitis found in the present study, 2.5/100,000 person-years, compared well with a study from Finland that found an increase from 2.4/100,000 in 1986 to 3.8/100,000 in 2000 [17] and two studies from UK that found incidence rates of 2.7/100,000 person-years between 1990 and 2005 [16] and 3.5/100,000 between 1995 and 2000 [27]. In the present study, most of the perforations, 89 %, occurred during the first admission for acute colonic diverticulitis. This was in accordance with other recent studies [19, 28, 29]. Elective resection of the sigmoid colon to prevent perforation after the first episode would be unnecessary for most patients, since the risk of free perforation is highest at the index episode [11, 30]. We are not able to identify the patients who present with perforation without prior symptoms of diverticulitis.

A recurrence rate of 25–30 % is usually reported [30–32], which compares well with that of the present study, although rates as low as 6.1 % have been reported [33]. Different ways to indicate recurrence, whether as an absolute rate or as an estimated rate using Kaplan-Meier analysis, may explain some of the reported differences. Moreover, in countries with higher incidence rates, one might also expect higher recurrence rates of this disease.

The relation between incidence rates of an acute disease in the population and admission rates to hospital for this disease is complex. Factors other than disease incidence may influence hospital admissions [34]. The threshold to seek medical examination, advice and treatment vary from person to person and may change with age, periods of time and geographical area. The decisive factor is likely the severity of the acute disease as perceived by the patient. Trivial infections require no treatment or can be managed by general practitioners, while more serious cases are admitted to hospital. The present study was limited to hospitalized patients.

One can argue that in recent years, a lower threshold for admission of less severe cases of acute diverticulitis might have led to the increased admission rates. This may in part be true. However, the study found that the median values of CRP in patients with uncomplicated diverticulitis increased significantly during the 25 years, both for values measured at admission as well as for the maximum measured values during the hospital stay. CRP varies with the severity of the diverticulitis and may aid to predict perforation [35]. The Poisson regression analyses showed a significant increase of admission rates during the 25 years, which could not be attributed only to an increased population or more elderly inhabitants.

A strong indication of a real increase in the incidence of acute diverticulitis in the population was the significant increase of the most severe forms of the disease, acute diverticulitis with perforation and purulent or faecal peritonitis. In our area, such a serious condition would result in referral to hospital, and it was also so during the early periods of the study. The incidence rate of perforated diverticulitis with peritonitis increased by a factor of 3.2 from 1988–92 to 2008–12; this compared well with the general increase of acute diverticulitis by a factor of 2.9. We expect that the change in incidence of perforated acute diverticulitis corresponds to a general increase in incidence of acute diverticulitis. On the other hand, the increase of diverticular perforations might partly be due to an increased use of NSAIDS, which is known to be associated with perforated diverticular disease [12].

### Weaknesses and limitations of the study

In a prospective study, a set of diagnostic tests and criteria can be implemented to ensure the diagnosis. In this retrospective study, we were not able to demand CT scan or other tests to confirm the diagnosis for every patient. Some cases might be missing if their discharge diagnosis codes had been intra-abdominal abscess or peritonitis, without also adding the diagnosis code for colon diverticulitis. We were not aware of a change in admission policy for acute diverticulitis during the study period. If more patients had been treated at home early in the study period, this might have affected the outcome of the study. Patients from this area were not included in the study if they had been treated for acute diverticulitis at another hospital while, for example, travelling. Likewise, patients who did not live here and were admitted for an acute abdominal condition at our hospital were included in the study.

### Strengths of the study

Our understanding of this disease [36] has been improved by the recent, extensive reports that have based their data on administrative data. The present study validated the administrative data by examining the case records of every patient, thus excluding patients with diverticular disease who did not have acute colonic diverticulitis.

Levanger Hospital serves a localized area with a relatively stable population, which makes this area suitable for epidemiological studies [37]. Patients with acute abdominal conditions, like acute diverticulitis, are admitted to one hospital that has had continuity in admission policy for acute diverticulitis during the 25 years of study. Because the patients in this series were recruited from a long period of time, this allowed assessment of trends, which might be difficult to discover within a shorter observation period. Very few studies have addressed this disease over such an extended period of time.

## Conclusions

This study has shown that the admission rates of diagnosed acute colonic diverticulitis increased significantly during the last 25 years. The incidence rates observed in hospital likewise increased. This most likely also reflected an increase in the incidence rates of acute diverticulitis in the population. The incidence increased with age, without an upper limit, and the disease may become even more challenging in the future with more people living longer.
